# The first organocatalytic carbonyl-ene reaction: isomerisation-free C-C bond formations catalysed by H-bonding thio-ureas

**DOI:** 10.1186/1860-5397-3-24

**Published:** 2007-09-14

**Authors:** Matthew L Clarke, Charlotte E S Jones, Marcia B France

**Affiliations:** 1 School of Chemistry, University of St. Andrews, EaStCHEM, St. Andrews, Fife, KY16 9ST, UK; 2 Department of Chemistry, Washington and Lee University, Lexington, VA 24450, USA

## Abstract

Intramolecular carbonyl ene reactions of highly activated enophiles can be catalysed by H-bonding thio-ureas to give tertiary alcohols in high yields without extensive isomerisation side products. An asymmetric variant of this reaction was realised using a chiral thiourea but was limited by low enantioselectivity (up to 33% e.e.) and low turnover frequencies.

## Background

The intermolecular carbonyl-ene reaction is a useful and completely atom-efficient C-C bond forming reaction. These reactions take place without a catalyst at relatively high temperatures (>150°C), but are more often carried out with either a stoichiometric or catalytic Lewis acid catalyst. [1–4] One of the drawbacks of the intermolecular carbonyl ene reaction is substrate scope. The majority of all successful catalytic ene reactions have utilised the highly activated glyoxylate esters as enophile. Extending asymmetric intramolecular carbonyl ene reactions to include ketone enophiles is a highly desired process that can potentially deliver enantio-enriched tertiary alcohols. The most promising results in this regard come from Evans and co-workers who demonstrated that 1,1-disubstituted alkenes would react with the activated ethyl pyruvate to give the desired tertiary alcohols in good yield. However, this reaction suffered from low turnover frequencies and was limited to certain types of alkene nucleophiles which were required in a large excess for the reaction to proceed.[5] However, a range of ketone ene reactions have been promoted by stoichiometric Et_2_AlCl, suggesting some potential for this reaction.[6] We have screened catalysts from across the periodic table with a wide range of ligands in an attempt to develop catalytic asymmetric ketone ene reactions of ketones of type RC(O)CO_2_Et with scant success. The ketones were unreactive, and the alkenes used often polymerised under the reaction conditions. An exception was the ene reaction of ethyl trifluoropyruvate **2** with alkenes which actually took place very readily, although sometimes accompanied by isomerisation and other side products. This type of ene reaction has recently been explored by other groups using Pd, Ni and Pt catalysts.[7–8] The importance of such reactions is derived from the ever-increasing occurrence of trifluoromethyl substituents in drugs and biologically active compounds.[9–10] Given that trifluoropyruvate ene reactions seemingly have a low activation barrier, and as part of our ongoing interest in hydrogen bond mediated catalysis,[11] we have investigated thioureas as H-bonding additives for organocatalytic carbonyl ene reactions and report these results here. Despite the explosive growth in organocatalytic reactions in recent years, [12–16] this represents, to the best of our knowledge, the first example of an organocatalytic carbonyl ene reaction. [17]

## Findings

**Scheme 1:**
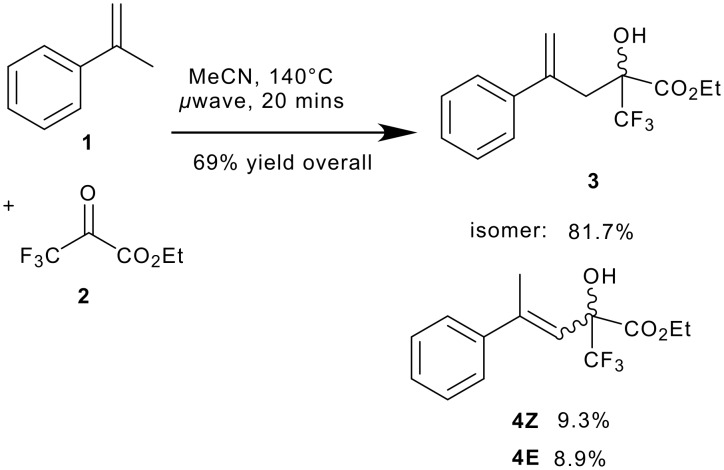
Microwave promoted ene reaction of ethyl trifluoropyruvate with α-methyl styrene.

We first carried out a thermal ene reaction of α-methyl styrene **1** with ethyl trifluoropyruvate under microwave heating to produce racemic product **3** (Scheme 1). This reaction was accompanied by several side products. The major one of these was clearly an (**E + Z**) alkene containing product. This was identified as isomerisation product **4** on the basis of ^19^F, ^1^H NMR spectroscopy and most informatively, GCMS of the isolated product mixture, which showed three species with the same mass but different retention times. Other minor side products were formed from the decomposition of ethyl trifluoropyruvate, but were readily separated from compounds **3** and **4**. This decomposition becomes more significant as the reaction is left longer.

**Scheme 2:**
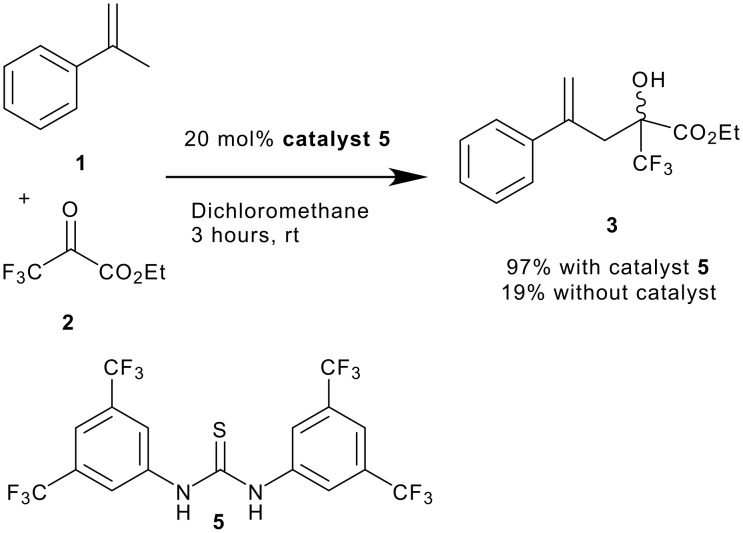
Thiourea catalysed ene reaction.

An investigation into the effect of various H-bonding additives on this reaction was carried out (Scheme 2 and Table 1). N,N'-di [3,5-bis(trifluoromethyl)phenyl]thiourea **5**,[18], emerged as by far the most active promoter for this reaction. The catalyst was used at 20 mol % loading and compared against a control reaction with no catalyst. Products were isolated by chromatography after 3 hours reaction time. A 96% yield of the desired isomerically pure tertiary alcohol was obtained in the presence of **5**. In contrast, the uncatalysed background reaction yields just 19%. Reactions monitored by ^19^F NMR reveal that good conversion (70%) was also possible after 1 hour using 10 mol % catalyst. Lower catalyst loadings required longer reaction times. Thus, in common with most organocatalytic procedures, turnover frequency is low.

**Table 1:** Effect of different thiourea on ene reaction.

**Thiourea**	**Time (hrs)**	**% Conv.** **^a^**

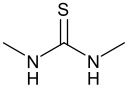	1	3
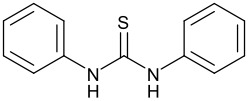	1	15
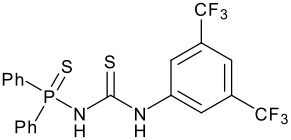	2	2
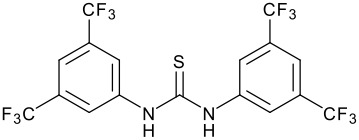	1	95

a: % Conversion determined by ^19^F {^1^H} NMR. Ethyl trifluoropyruvate (0.098 ml, 0.75 mmol) was added to a solution of the thiourea (20 mol%) and the α-methyl styrene (0.12 ml, 0.9 mmol, ~1.2 eq) in dichloromethane (2 ml).

**Scheme 3:**
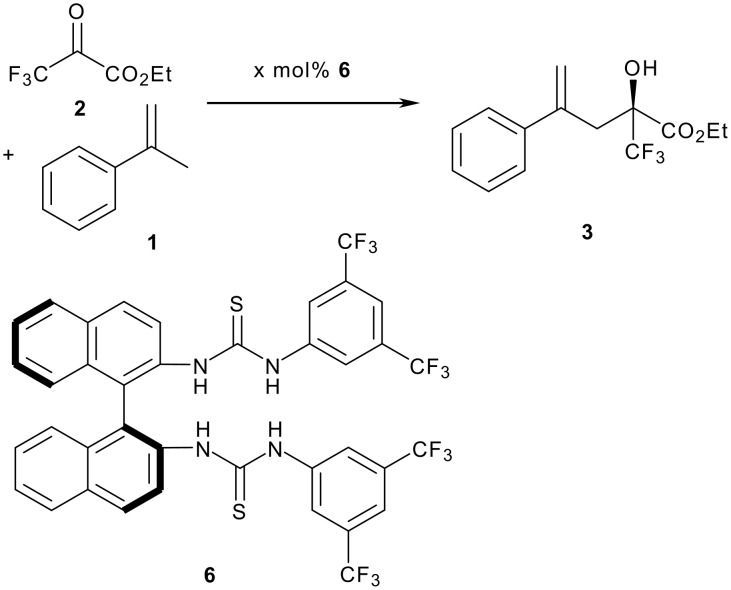
Asymmetric carbonyl ene reaction mediated by chiral thiourea.

In order to explore if an asymmetric variant of this reaction was possible, we elected to employ a chiral thio-urea. It is almost certain that these reactions proceed by H-bonding of the ketone to the thio-urea moiety, lowering the LUMO for attack by uncomplexed alkene. An asymmetric catalyst would therefore need to be a *diaryl* thio-urea in order to maximise the acidity of the NH functions. It would also need to possess chirality that can potentially close off one face of the co-ordinated ketone that would also have to hydrogen bond in a defined manner. The most readily available thio-urea that could hopefully fulfil these two roles is thio-urea **6**, which was prepared by reaction of (R)-(+)-1,1'-Bi(2-naphthylamine) with 3,5-bis(trifluoromethyl)phenyl isothiocyanate in 52% yield and has recently been reported by another group.[19] This catalyst was tested in the α-methyl styrene ene reaction to ethyl trifluoropyruvate with ultimately disappointing results. This catalyst did allow us to demonstrate the first asymmetric organocatalytic carbonyl ene reaction, but reactions run at both -20 and 0°C using 10 and 25 mol % catalyst respectively gave good yields and only ~30% e.e (Scheme 3 and Table 2). A reaction carried out with stoichiometric amounts of chiral thiourea also gave ~30% e.e. suggesting this is the true selectivity for this reaction with this catalyst (and not complicated by an unselective background reaction).

**Table 2:** Asymmetric ene reaction.

**X mol% 6**	**Temp (°C)**	**Time (hrs)**	**Yield**	e.e.*

10	0	16	32	23
10	-20	210	89	33
25	-20	345	65	30
25	0	90	70	26
100	-20	46	67	28

*Enantioselectivity determined using Eu(hfc)_3_ and ^19^F NMR Ethyl trifluoropyruvate (0.098 ml, 0.75 mmol) was added to a solution of the thiourea (x mol%) and the α-methyl styrene (0.12 ml, 0.9 mmol, ~1.2 eq) in dichloromethane (2 ml) and left for the time shown before purification by column chromatography.

**Scheme 4:**
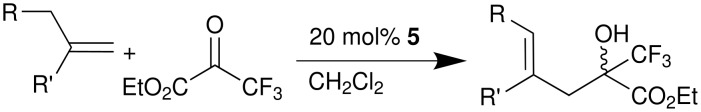
Reaction between ethyl trifluoropyruvate and various alkenes.

Before investigating any other asymmetric catalysts, the scope of the organocatalytic ene reaction was examined briefly. The reactions was found to proceed readily with 1,1'disubstituted alkenes, but was less effective with terminal alkenes which gave lower yields (Scheme 4, Table 3 and Supporting Information File 1). We note here that the ene reactions of the highly activated substrates proceed well without catalyst at room temperature: testament to trifluoropyruvate being a highly activated enophile. Catalyst **5** also did not catalyse ene reactions between α-methylstyrene and ethyl glyoxylate, trifluoro-acetophenone, or α-bromopyruvate. However, we have not had success with transition metal catalysed ketone ene reactions using these latter two substrates either. These studies reveal that the scope of the organocatalytic reactions is severely limited. A step change in catalytic performance is required for this reaction to truly reach its potential. It remains to be seen whether the more entropically favoured intramolecular carbonyl ene cyclisation reaction holds more promise.

**Table 3:** Investigation of different alkenes in the ene reaction of trifluoropyruvate

	**Alkene** **^a^**	**Time (hrs)**	**% conversion catalysed reaction**	**% conversion uncatalysed reaction**

1	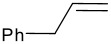	16	18	0
2	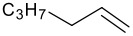	18	22	3
3	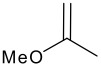	1	94	95
4	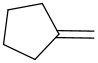	1	95	93

A: Ethyl trifluoropyruvate (0.098 ml, 0.75 mmol) was added to a solution of the thiourea **5** (75 mg, 20 mol%) and the α-methyl styrene (0.12 ml, 0.9 mmol, ~1.2 eq) in dichloromethane (2 ml).

In summary, we have developed the first organocatalytic carbonyl ene reaction and shown that some asymmetric induction is possible if a chiral thiourea catalyst is employed. However, turnover frequency, enantioselectivity and substrate scope are all very modest, suggesting that for *intermolecular* ene reactions, organocatalysis may not be a promising approach. Further work on intramolecular organocatalytic ene reactions is required, which may provide synthetically useful reactions.

## Supporting Information

File 1 first organocatalytic carbonyl ene supporting info. Experimental procedures and spectral data for the products.

## References

[1] Snider B B (1980). Acc Chem Res.

[2] Mikami K, Shimizu M (1992). Chem Rev.

[3] Dias L C (2000). Curr Org Chem.

[4] Berrisford D J, Bolm C (1995). Angew Chem, Int Ed Engl.

[5] Evans D A, Tregay S W, Burgey C S, Paras N A, Vojkovsky T (2000). J Am Chem Soc.

[6] Jackson A C, Goldman B E, Snider B B (1984). J Org Chem.

[7] Aikawa K, Kainuma K, Hatano M, Mikami K (2004). Tetrahedron Lett.

[8] Doherty S, Knight J G, Smyth C H, Harrington R W, Clegg W L (2006). J Org Chem.

[9] Isanbor I, O'Hagan D (2006). J Fluorine Chem.

[10] Mizuta S, Shibata N, Hibino M, Nagano S, Nakamura S, Toru T (2007). Tetrahedron.

[11] Clarke M L, Fuentes J A (2007). Angew Chem, Int Ed.

[12] Clarke M L (2004). Lett Org Chem.

[13] Dalko P I, Moisan L (2004). Angew Chem, Int Ed.

[14] Seayad J, List B (2005). Org Biomol Chem.

[15] Taylor M S, Jacobsen E N (2006). Angew Chem, Int Ed.

[16] Connon S J (2006). Angew Chem, Int Ed.

[17] Gotoh H, Masui R, Ogino H, Shoji M, Hayshi Y (2006). Angew Chem, Int Ed.

[18] Witkopp A, Schreiner P R (2003). Chem–Eur J.

[19] Fleming E M, McCabe T, Connon S J (2006). Tetrahedron Lett.

