# Supplementary material for: The first organocatalytic carbonyl-ene reaction: isomerisation-free C-C bond formations catalysed by H-bonding thio-ureas
Source: Beilstein J Org Chem. 2007 Sep 14;3:24. doi: 10.1186/1860-5397-3-24 (PMC2176055; doi:10.1186/1860-5397-3-24)
Supplement: File 1 — first organocatalytic carbonyl ene supporting info. Experimental procedures and spectral data for the products. [file Beilstein_J_Org_Chem-03-24-s001.pdf]

The first organocatalytic carbonyl-ene reaction: isomerisation-free C-C bond formations catalysed by H-bonding thio-ureas.

Charlotte E. S. Jones,[1] Matthew L. Clarke*[1] and Marcia B. France.[2]

Supporting information

***General information*:** All chemicals and solvents are standard laboratory grade, obtained from commercial sources and were used as received. Dry, degassed solvents were used for reactions unless otherwise indicated. Normal grade solvents were used for chromatography. Solvents were removed by rotary evaporation on a Heidolph labrota 4000. Flash column chromatography was performed using Davisil silica gel Fluorochem 60Å, particle size 35-70 micron. All NMR spectra were recorded on Bruker Avance 300 instruments. Mass spectra were recorded on Water Micromass GCT (Time of flight) fitted with lockspray for accurate mass (ESI) or GCT (CI) instruments. All known compounds gave the expected mass spectra. Microanalyses (CHN) were carried out at the University of St. Andrews and are quoted to the nearest 0.05 percent. Infra-red (IR) spectra were recorded on NaCl plates using a Perkin-Elmer Spectrum GX spectrometer. In the assignment of ^{13}C NMR the symbols quat, CH, CH_2 and CH_3 are used to denote a quaternary, primary, secondary and tertiary carbon centres respectively. In the assignment of all NMR spectra the abbreviation Ar is used to denote aromatic.

Microwave reactions were carried out in a Biotage® Initiator using 10 ml heavy-walled reactor vials equipped with an air tight seal. The temperature is measured by an infra red temperature probe that measures the temperature on the surface of the vial. The pressure is measured by direct reading of the deflection of the septa on the vial using a load cell behind the inner part of the cavity lid.

Catalyst Preparation

Synthesis of N,N'-di[3,5-di(trifluoromethyl)phenyl]thiourea

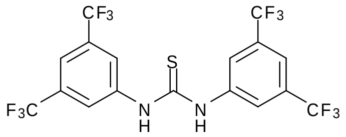

3,5-bis(trifluoromethyl)phenylisothiocyanate (1.83 ml, 10 mmol) was added to a solution of 3,5-bis(trifluoromethyl)aniline (1.63 ml, 10.5 mmol) in dichloromethane (5 ml) at 0°C under nitrogen. The mixture was left to stir for 10 minutes then a further 18 hours at room temperature. The solvent was removed under reduced pressure and the residue washed with cold dichloromethane to give a colourless solid (3.6g, 72%).

υ_{max}(KBr)/cm^{-1} 3170, 2920, 1555, 3207, 1467, 1376, 1179, 1139, 929, 890; δ_H(300MHz, d6-DMSO) 10.64 (2 H, s, NH), 8.19 (4 H, s, Ar-H), and 7.86 (2 H, s, Ar-H); δ_F(282 MHz, d6-DMSO) -61.94; δ_C(75.4 MHz, $CDCl_3$) 180.5 (quat, C=S), 141.1 (quat, Ar), 130.3 (quat, J_{CF} 33.0, Ar), 124.0 (CH, Ar), 123.0 (quat, J_{CF} 272.7, CF_3), 117.5 (CH, Ar); m/z (ES -ve) 499.96 (29%, M-H), 498.88 (100%).

Witkopp A, Schreiner PR: *Chem Eur—J* 2003, **9**: 407

Synthesis of diphenylphosphinothioyl chloride

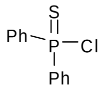

Sulfur (1.78 g, 55 mmol) was added to a solution of chlorodiphenylphosphine (2.6 ml, 13.9 mmol) in dry degassed toluene (7 ml). The mixture was heated under nitrogen at 105ºC for 4.5 hours. The mixture was cooled to room temperature and filtered. The solvent was removed under reduced pressure and the residue redissolved in dry degassed ether (8ml). The mixture was cooled to 0ºC overnight, filtered and the solvent removed under reduced pressure to give a pale yellow oil (3.48 g, 91%).

δ_H(300MHz, $CDCl_3$) 7.92-7.83 (2H, m, Ar-H), 7.49-7.39 (8H, m, Ar-H); δ_P(121.4 MHz, $CDCl_3$) 81.3.

Spence RA, Swan JM, Wright SHB: *Australian J Chem* 1969, **22**: 2359

Synthesis of diphenylphosphinothioyl isothiocyanate

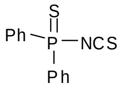

Potassium isothiocyanate (4.12 g, 42.3 mmol) was added to a solution of diphenylphosphinothioyl chloride (3.46 g, 13.7 mmol) in degassed acetonitrile (30 ml). The mixture was heated to 95ºC for 1.5 hours under nitrogen and then filtered. The solvent was removed from the filtrate under reduced pressure to give a yellow solid. This was further purified by dissolving the residue in dry pentane and filtering the mixture. The filtrate was again concentrated to give the product, a pale yellow solid (2.86g, 76%).

δ_H(300MHz, $CDCl_3$) 7.86-7.74 (2H, m, Ar-H), and 7.53-7.38 (3H, m, Ar-H); δ_P(121.4 MHz, $CDCl_3$) 57.5, δ_C(75.4 MHz, $CDCl_3$) 149.3 (quat, C=S), 132.5 (quat, J_{CP} 77.7, Ar), 131.6 (CH, J 3.2, Ar), 129.6 (CH, J_{CP} 12.3, Ar), 127.8 (CH, J_{CP}14.3, Ar).

Spence RA, Swan JM, Wright SHB: *Australian J Chem* 1969, **22**: 2359

Synthesis of 1-(3,5-bis(trifluoromethyl)phenyl)-3-(diphenylphosphorothioyl)thiourea

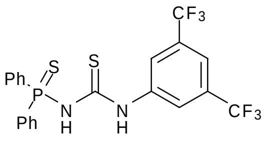

3,5-bis(trifluoromethyl)aniline (0.61 ml, 3.94 mmol) was added to a solution of diphenylphosphinothioyl isothiocyanate (1 g, 3.67 mmol) in degassed tetrahydrofuran (7ml). The mixture was left to stir under nitrogen for a week until the reaction had gone to completion by ^{31}P NMR. The solvent was removed *in vacuo* and the residue recrystallised in dichloromethane to give a colourless solid (1.11g, 62%). This compound has not been reported previously.

mp 120-122°C υ_{max}($CDCl_3$)/cm^{-1} 2920, 1734, 1629, 1583, 1461, 1140, 997, 680, 635; δ_H(300MHz, $CDCl_3$) 10.89 (1H, br s, NH), 7.89 (1H, s, Ar), 7.82 (4H, d, J 7.02Hz, 7.61-7.46 (6H, m, Ar) and 7.03 (1H, br s, NH); δ_F(282 MHz, $CDCl_3$) -64.2; δ_P(121.4 MHz, $CDCl_3$) 54.2; δ_C(75.4 MHz, d6-DMSO) 150.0 (quat, *C*=S), 134.8 (quat, J_{CP}134.7, Ar), 131.4 (CH, J_{CP} 2.4, Ar), 130.8 (CH, J_{CP} 9.9, Ar), 130.8 (quat, J_{CF} 32.1, Ar), 128.4 (CH, J_{CP} 12.5, Ar), 123.6 (quat, J_{CF} 272.4, CF_3), 113.0 (CH, Ar) and 107.4 (CH, Ar); m/z (ES) 536.2 (M+MeOH) (Found: C, 50.15; H, 2.75; N, 5.45. $C_{21}H_{15}N_2F_6PS_2$ requires C, 50.00; H, 3.00; N 5.55%).

Synthesis of N,N''-(1R)-[1,1'-binaphthalene]-2,2'-diylbis[N'-[3,5-bis(trifluoromethyl)phenyl]-thiourea

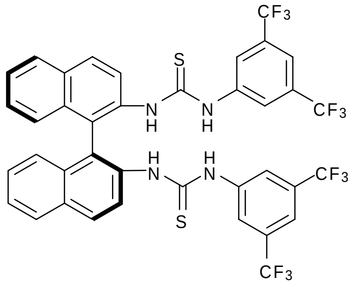

(R)-2,2'-diamino-1,1'-binapthalene (127 mg, 0.44 mmol) was transferred under an argon blanket and dissolved in dry THF (2 ml). 3,5-bis(trifluoromethyl)phenyl isothiocyanate (0.16 ml, 0.89 mmol, ~2 eq.) was added and the mixture was left to stir for 16 hours at room temperature. Dichloromethane was added (10 ml) and the solvent was removed under reduced pressure. The reaction mixture was further purified by column chromatography eluting with 9:1→5:1 hexane: ethyl acetate to give a pale yellow solid (122 mg, 52%).

υ_{max}(KBr)/cm^{-1} 3156 (NH), 2930 (CH), 1701, 1619, 1177, 886; δ_H(300 MHz; $CDCl_3$) 8.07 (2 H, d, J 8.7, Ar-H), 7.94 (2 H, s, Ar-H), 7.89 (2 H, d, J 8.19, Ar-H), 7.80 (2 H, d, J 8.96, Ar-H), 7.63 (4 H, s, Ar-H), 7.54 (2 H, s, NH), 7.50 (2 H, s, NH), 7.43 (2 H, t, J 6.91, Ar-H), 7.22 (2 H, t, J 8.19, Ar-H), and 7.04 (2 H, d, J 9.21, Ar-H);δ_F(282 MHz, $CDCl_3$) -63.5; δ_C(75.4 MHz, $CDCl_3$) 180.07 (quat, C=S), 138.7 (quat, Ar), 133.5 (quat, Ar), 132.7 (quat, Ar), 132.4 (quat, Ar), 131.9 (quat, J_{CF} 33.8, Ar), 130.7 (CH, Ar), 128.8 (CH, Ar), 128.1 (CH, Ar), 127.5 (quat, Ar), 127.1 (CH, Ar), 125.4 (CH, Ar), 124.8 (CH, br, Ar), 124.2 (CH, Ar) and 122.7 (quat, J_{CF} 272.9, CF_3); m/z (ES) 849.25 (100%, M+Na).

E. M. Fleming, T. McCabe, and S. J. Connon, *Tetrahedron Lett.*, 2006, **47**: 7037.

Example of ene reaction in microwave: Synthesis of ethyl 2-hydroxy-4-phenyl-2-(trifluoromethyl)pent-4-enoate and its isomers

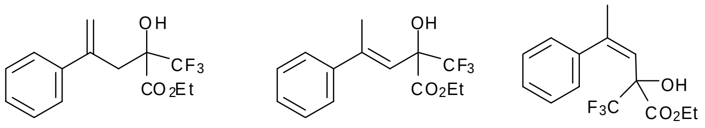

Ethyl trifluoropyruvate (0.098 ml, 0.75 mmol) was added to a solution of the olefin (0.9 mmol, ~1.2 eq) in dichloromethane (2 ml) under an inert atmosphere. The mixture was heated to 140ºC for 30 minutes. The solvent was removed *in vacuo* and the crude product purified by chromatography eluting with 6:1 hexane: ethyl acetate to give the product as a colourless oils. The isomers 3, 4E and 4Z were not separable and characterised by NMR and GCMS.

63% isolated yield from reaction, 56% **3**, GC-EIMS **4** 13.78 min (288.12, M^+), δ_F(282 MHz, $CDCl_3$) -79.7; **3** 14.08 mins (288.12, M^+), δ_F(282 MHz, $CDCl_3$) -79.5; **4** 14.96 mins (288.12, M^+) δ_F(282 MHz, $CDCl_3$) -79.5.

Procedure for testing of thioureas in ene reaction ethyl trifluoropyruvate with alkene

Ethyl trifluoropyruvate (0.098 ml, 0.75 mmol) was added to a solution of the thiourea and the alkene (0.9 mmol, ~1.2 eq) in dichloromethane (2 ml). The mixture was left to stir until analysis by ^{19}F NMR at which point it was transferred to an NMR tube under nitrogen with a d6-benzene filled capilliary for analysis. The percentage conversion was taken from the shift integration of the product over the total integration of fluorine peaks. Product peaks were confirmed by spiking experiments with authentic samples. Pure products were isolated by column chromatography and characterised as described below.

Selected NMR

Ethyl 2-hydroxy-4-phenyl-2-(trifluoromethyl)pent-4-enoate

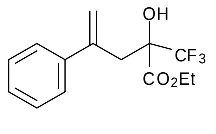

δ_H(300MHz, $CDCl_3$) 7.32-7.19 (5 H, m, Ar-H), 5.31 (1 H, s, C=C*H*H'), 5.21 (1 H, s, C=CH*H'*), 4.02-3.90 (1 H, m, C*H*H'), 3.70 (1 H, s, OH), 3.61-3.49 (1 H, m, CH*H'*), 3.21 (1 H, d, J 13.98, C*H*H'), 2.96 (1 H, d, J 13.98, CH*H'*), and 1.08 (3 H, t, J 7.16, CH_3); δ_F (282 MHz, $CDCl_3$) -78.9; δ_C(75.4 MHz, $CDCl_3$) 168.9 (quat, C=O), 141.1 (quat, C=C), 128.2 (CH, Ar), 127.7 (CH, Ar), 126.8 (CH, Ar), 123.4 (quat, J_{CF} 286.3, CF_3), 119.4 (quat, Ar), 77.1 (quat, J_{CF} 28.9), 63.5 (CH_2), 37.0 (CH_2) and 22.1 (CH_3).

(E)-ethyl 2-hydroxy-2-(trifluoromethyl)oct-4-enoate

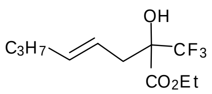

δ_H(300 MHz, $CDCl_3$) 5.59-5.47 (1 H, m, C=CH), 5.30-5.19 (1 H, m, C=CH), 4.26 (2 H, q, J 6.65, CH_2), 3.72 (1 H, s, OH), 2.65-2.47 (2 H, m, CH_2), 1.90 (2 H, q, J 7.08, CH_2), 1.35-1.22 (5 H, m), and 0.78 (3 H, t, J 7.08, CH_3);δ_F(C282 MHz, DCl_3) -78.9. δ_C(75.4 MHz, $CDCl_3$) 169.4 (CO), 136.5 (CH) 123.4 (quat, J_{CF} 286.2), 121.3 (CH) 77.8 (quat, J_{CF} 28.4) 62.1 (CH_2), 36.2(CH_2), 29.9 (CH_2), 20.4(CH_2), 13.5 (CH_3) and 12.9 (CH_3).

Mikami K, Aikawa K, Kainuma S, Kawakami Y, Saito T, Sayo N, Kumobayashi H, *Tetrahedron Asymmetry* 2004, **15**: 3885

(E)-ethyl 2-hydroxy-5-phenyl-2-(trifluoromethyl)pent-4-enoate

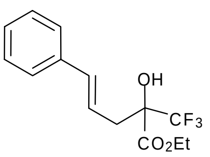

δ_H(300MHz, $CDCl_3$) 7.27-7.20 (5 H, m, Ar-H), 6.46 (1 H, d, J 16.59, C=CH), 6.00 (1 H, m, C=CH), 4.27 (2 H, m, CH_2), 2.79 (2 H, m, CH_2), and 1.26 (3 H, t, J 7.14, CH_3); δ_F(282 MHz, $CDCl_3$) -78.81, δ_C(75.4 MHz, $CDCl_3$) 169.3 (quat, CO), 136.6 (quat, Ar), 135.5 (CH, Ar), 128.6 (CH, Ar), 127.8 (CH, Ar), 126.3 (CH, Ar), 123.4 (quat, J_{CF} 286.4, CF_3), 120.4 (CH, Ar), 77.4 (quat, J_{CF} 35.3, Ar), 63.9 (CH_2, CO_2CH_2), 35.4 (CH_2, $CH_2C(OH)$), 14.1 (CH_3).

Mikami K, Aikawa K, Kainuma S, Kawakami Y, Saito T, Sayo N, Kumobayashi H, *Tetrahedron Asymmetry* 2004, **15**: 3885

Ethyl 2-(cyclopentenylmethyl)-3,3,3-trifluoro-2-hydroxypropanoate

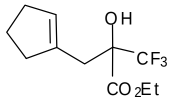

δ_H(300MHz, $CDCl_3$) 5.46 (1 H, s, C=CH), 4.32-4.21 (2 H, m, CH_2), 3.72 (1 H, s, OH), 2.79 (1 H, d, J 4.37, C*H*H'), 2.60 (1 H, d, J 4.40, CH*H'*), 2.34-2.06 (4 H, m, CH_2), 1.76 (2 H, quintet, J 7.27, CH_2), and 1.26 (3 H, t, J 7.14, CH_3); δ_F(282 MHz, $CDCl_3$) -79.18; δ_C(75.4 MHz, $CDCl_3$) 169.8 (quat, CO), 136.4 (quat, *C*=CH), 130.1 (CH, *C*H=C), 123.3 (quat, J_{CF} 286.4, CF_3), 77.8 (quat, J_{CF} 29, *C*-OH), 63.7 (CH_2,OCH_2CH_3), 36.0 (CH_2, $C(OH)CH_2$), 33.0 (CH_2, cy), 32.5 (CH_2, cy), 23.6 (CH_2, cy), and 13.9 (CH_3).

Mikami K, Aikawa K, Kainuma S, Kawakami Y, Saito T, Sayo N, Kumobayashi H, *Tetrahedron Asymmetry* 2004, **15**: 3885

Characterisation of ethyl 2-hydroxy-4-methoxy-2-(trifluoromethyl)pent-4-enoate

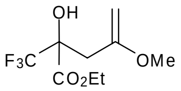

υ_{max}(film)/cm^{-1} 3476 (OH), 2895(CH), 1751 (CO), 1370, 1231, 1190, 1146, 863, 701. δ_H(300MHz, $CDCl_3$) 4.26 (2 H, q, J 7.14, CH_2), 4.00 (2 H, m), 3.89 (1 H, s, C=C*H*), 3.40 (3 H, s, CH_3), 2.86 (1 H, d, J 14.13, C*H*H'), 2.58 (1 H, d, J 14.13, CH*H'*), and 1.25 (3 H, t, J 14.16, CH_3); δ_F(282 MHz, $CDCl_3$) -79.0; δ_C(75.4 MHz, $CDCl_3$) 169.0 (quat, C=0), 156.0 (quat, C=C), 123.3 (quat, J_{CF} 286.1, CF_3), 86.0 (CH_2, C=CH_2), 77.8 (quat, J_{CF} 32.4, Ar), 63.4 (CH_2), 54.9 (CH_3), 37.7 (CH_2), and 13.8 (CH_3); *m/z* (CI) 243.0863 (MH^+ $C_9H_{14}O_4F_3$ requires 243.0844).
